# Wearable Cardiorespiratory Sensors for Aerospace Applications †

**DOI:** 10.3390/s22134673

**Published:** 2022-06-21

**Authors:** Nichakorn Pongsakornsathien, Alessandro Gardi, Yixiang Lim, Roberto Sabatini, Trevor Kistan

**Affiliations:** 1 School of Engineering, RMIT University, Bundoora, VIC 3083, Australia; keba@gmail.com (N.P.); malipero@gmail.com (T.K.); 2 Department of Aerospace Engineering, Khalifa University of Science and Technology, Abu Dhabi 127788, United Arab Emirates; jeaa@gmail.com; 3 School of Engineering, RMIT University, Melbourne, VIC 3001, Australia; 4 Saab-NTU Joint Lab, Nanyang Technological University, Singapore 639798, Singapore; zilecar@gmail.com; 5 THALES Australia—Airspace Mobility Solutions, Melbourne, VIC 3000, Australia

**Keywords:** Air Traffic Management, cognitive ergonomics, cardiorespiratory, ECG, fuzzy systems, heart rate, mental workload

## Abstract

Emerging Air Traffic Management (ATM) and avionics human–machine system concepts require the real-time monitoring of the human operator to support novel task assessment and system adaptation features. To realise these advanced concepts, it is essential to resort to a suite of sensors recording neurophysiological data reliably and accurately. This article presents the experimental verification and performance characterisation of a cardiorespiratory sensor for ATM and avionics applications. In particular, the processed physiological measurements from the designated commercial device are verified against clinical-grade equipment. Compared to other studies which only addressed physical workload, this characterisation was performed also looking at cognitive workload, which poses certain additional challenges to cardiorespiratory monitors. The article also addresses the quantification of uncertainty in the cognitive state estimation process as a function of the uncertainty in the input cardiorespiratory measurements. The results of the sensor verification and of the uncertainty propagation corroborate the basic suitability of the commercial cardiorespiratory sensor for the intended aerospace application but highlight the relatively poor performance in respiratory measurements during a purely mental activity.

## 1. Introduction

In complex missions dealing with large amounts of information in time-critical situations, such as in the case of Air Traffic Management (ATM), human operators need to work with high levels of automation support to improve operational performance. Dynamically adaptive Human–Machine Interfaces and Interactions (HMI^2^) have the potential to modulate the cognitive load, supporting increased autonomy in decision support systems [1,2]. Several researchers proposed the use of neurophysiological monitoring to drive HMI^2^ adaptation [3]. The main physiological observables that can be monitored include brain [4], cardiorespiratory [5], and eye [6] activity, though research is also addressing facial expression [7,8] and voice pattern analysis [9,10,11,12] to estimate the human operator’s cognitive states. Each physiological observable provides a diverse perspective on the physical and mental state of the monitored human and has a different level of intrusiveness and ergonomics impact [13]. In metrological terms, some sensors are faster but more susceptible to Electro-Magnetic Interference (EMI), while others are slower but more resilient to noise and disturbances. For these important reasons, there is considerable research interest in various neurophysiological observables and associated sensing technologies for the aerospace sector.

Recent research has characterised the performance of eye tracking sensors [14], which showed great promise for avionics and ATM applications. Cardiorespiratory sensors are simpler and have more clinical heritage than Electro-Encephalography (EEG) and eye-tracking. They are also less susceptible to interference and several cardiorespiratory monitors are much less intrusive (and obtrusive) compared to EEG. In this article, we therefore focus on the application of cardiorespiratory sensors to support adaptive HMI^2^ in aviation. While the performance of cardiorespiratory sensor technology has been widely studied, notable works only addressed physical activity [15,16,17,18,19,20,21,22,23,24,25]. A number of studies have looked at utilizing cardiorespiratory observables for cognitive state estimation in the air traffic control context [26,27]. However, the quality of data, which depends on the actual performance of the sensors, needs to be assessed to ensure repeatable and conclusive findings [28]. Therefore, for more complex aviation tasks, it is essential to characterise the performance of Electrocardiographic (ECG) sensors by comparing the correlation of cardiorespiratory features against objective measures such as task performance. ECG sensors were used in flight applications to estimate mental workload of the pilot [29,30,31], but the medical-grade devices used in these studies are typically not suitable for real-time applications due to their high level of intrusiveness and lack of support for real-time data sharing. However, the emergence of new consumer-grade devices in the market offers great promise for adaptive HMI^2^ applications as they have the ability to measure cardiorespiratory observables with relatively high accuracy while overcoming the aforementioned shortcomings.

Significant aerospace research focusses on consumer-grade, wearable sensors, for which there are a number of published studies as reviewed in [23]. None of these studies followed a mental testing protocol and the wearable cardiorespiratory monitoring device featured in these studies was neither monitoring mental Workload (WL) nor used for aerospace human factors purposes. This lack of pre-experiment characterisation and performance analysis is particularly critical considering the significant body of research featuring these sensors in aviation [26,27,29,30,31,32]. Because of this growing adoption of cardiorespiratory monitoring in aerospace human factors research, it is of paramount importance to investigate their performance in cognitively complex tasks.

This article addresses the verification and performance characterisation of commercial cardiorespiratory sensors in monitoring cognitive tasks, focussing on aerospace applications. The paper is a substantially extended version of the initial work presented in [33]. In addition to extending the characterisation beyond the sole cardiac measurements, this article introduces a strategy to apply the classic propagation of uncertainty theory through a machine learning classifier, allowing to determine the uncertainty in the final cognitive state estimations. Because of the limited experimental data available in the literature, this paper does not claim to conduct a statistically representative analysis, but instead to complete a preliminary verification in a mental workload setting and to propose and demonstrate a new approach to uncertainty propagation through neuro-fuzzy inference systems. Both of these aspects are novel and important for the research conducted in Air Traffic Management (ATM) around the world.

### Cardiorespiratory Sensing in the CHMI^2^ Framework

Spikes in WL in human pilot and ATM operator roles are particularly hazardous and therefore are being addressed by a number of ongoing human factors engineering studies [3]. A key objective for most of these studies is the development of HMI^2^ which not only allows the operator to maintain a better awareness of the system’s actions, but also prevents cognitive overload and hazardous instances, including attention tunnelling or being left “out-of-the-loop”. One emerging concept holding significant promise to enhance operational safety and efficiency optimisation is termed Cognitive HMI^2^ (CHMI^2^), where a system senses the cognitive state of the human operator and dynamically adapts HMI^2^ formats to provide real-time support [34,35].

**Figure 1:**
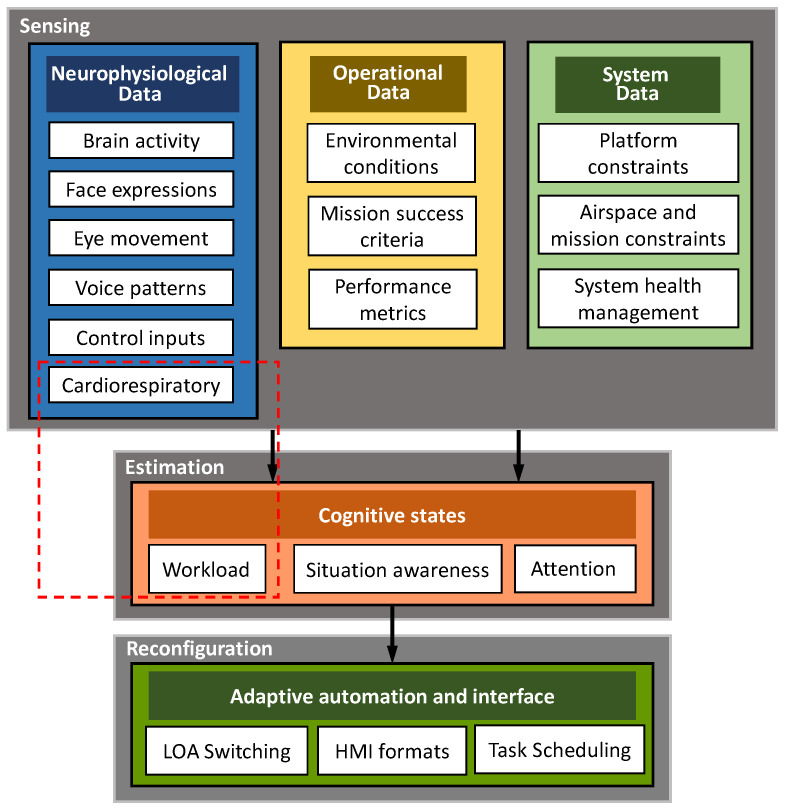
Top-level architecture of the CHMI^2^ system.

The CHMI^2^ cognitive state estimation algorithms receive input data from a combination of wearable and stand-off biosensors and from other avionics systems and data sources and convert these physiological, operational, and environmental variables into cognitive states exploiting a machine-learning based classifier which was previously trained on the particular human operator following a specifically designed pattern. Figure 1 shows the top-level CHMI^2^ system architecture tailored for adaptation of: Level of Autonomy (LOA), Human–Machine Interface (HMI) formats, and ATM task scheduling. A detailed description of the CHMI^2^ system with a focus on the currently adopted neurophysiological sensor network is provided in [13].

The real-time sensing of cardiorespiratory parameters is important in the CHMI^2^ system because, among other states, these have shown to provide an accurate indication of the level of effort (either mental or physical) of the subject. Moreover, they have been studied for a considerable time, so a significant body of literature is available, and the sensing technology is mature. One notable practical disadvantage is that both cardiac and respiratory responses have a relatively low temporal sensitivity (lagging around four to six seconds) [36] compared to other physiological signals (eye-based parameters have a relatively higher temporal sensitivity in the order of milliseconds [37]) which leads to inaccuracies during fusion of different physiological features, therefore a careful mapping of stimulus time with physiological responses is also needed.

## 2. Models and Methods

**Figure 2:**
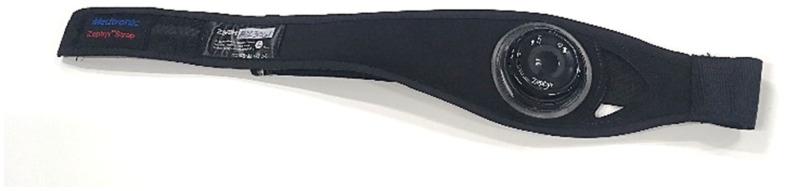
Commercial device adopted for the ATM CHMI^2^ research.

The consumer-grade sensor under investigation is the Zephyr BioHarness (BH) shown in Figure 2, which is widely available commercially and frequently used for activity monitoring (particularly of the physical type). Such a device, henceforth referenced as *commercial device*, also serves as a good reference for similar wearable consumer-grade devices. The BH is a lightweight chest-mounted sensor with a chest strap weighing 71 g and a processing module weighing 18 g that logs and transmits the data in real time. It can measure five physiological parameters simultaneously: Heart Rate (HR), Breathing Rate (BR), skin temperature, tri axial accelerometery, and posture. BH reports frequencies of 250 Hz for the ECG waveform and 18 Hz for the breathing waveform. These raw measurements are processed into HR, HRV, and BR indicators, which are logged or streamed either upon detected variations or at predefined intervals, with the most common choices being 1 or 2 Hz. The literature suggests that these sampling frequencies are adequate for HR, HRV, and BR indicators as these are only defined as a function of the peak-to-peak interval (e.g., RtoR) in the raw signal, which for adult non-athletic participants lies between 0.3 and 4 Hz [38]. The technical documentation provided by the manufacturer quotes that readings can deviate ±2 bpm for heart rate and ±3 bpm for breathing rate in low activity or static mode. The cardiorespiratory data measured from BH includes raw Electrocardiogram (ECG) signal (electric potential), raw respiratory waveform, as well as processed HR and BR. The BH determines HR by capturing cardiac electrical impulses for electronic filtration and analysis by conductive silver-coated nylon skin electrodes, which are subsequently relayed to the transmitter. For BR, a strain gauge is exploited, so that the conductor’s resistance increases proportionally to the length of the conductive fabric, flexible Mylar, and foam. This variation in resistance is measured using a proprietary capacitive sensor. The chest expansion and contraction lead to size differentials that induce capacitance changes due to impedance changes. The waveform of such variations is recorded [39]. For processed HR signal, high-pass and low-pass filters are applied on the raw ECG with 15 Hz and 78 Hz cut-off frequencies, respectively [40]. These cut-off frequencies allow measurement of HR under vigorous activity.

**Figure 3:**
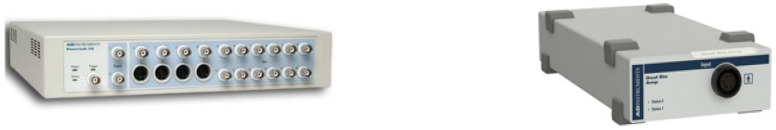
PowerLab 8/30 with Dual BioAmp DB066 unit (image by ADInstrument, reproduced with permission).

The measurements from the commercial device are compared with the data from a medical-grade and clinically-validated ECG equipment. Both sensors are used simultaneously under rest condition to determine baseline measurements and higher MWL conditions, allowing direct one-to-one comparison. An algorithmic synchronization is implemented in the data logging routine for both sensors to ensure optimal consistency between the BR rates of both instruments. The clinical ECG equipment used in our study is the ADInstrument PowerLab 8/30 with Dual BioAmp DB066 unit (Figure 3), which is widely used in medical research applications [41]. As the performance of this device is well verified, data from this sensor can be used as the reference datum for the verification of the commercial device. The sampling rate for each ADI ECG signal channel is 1000 Hz and the individual ECG channels are recorded and stored using the LabChart software.

**Figure 4:**
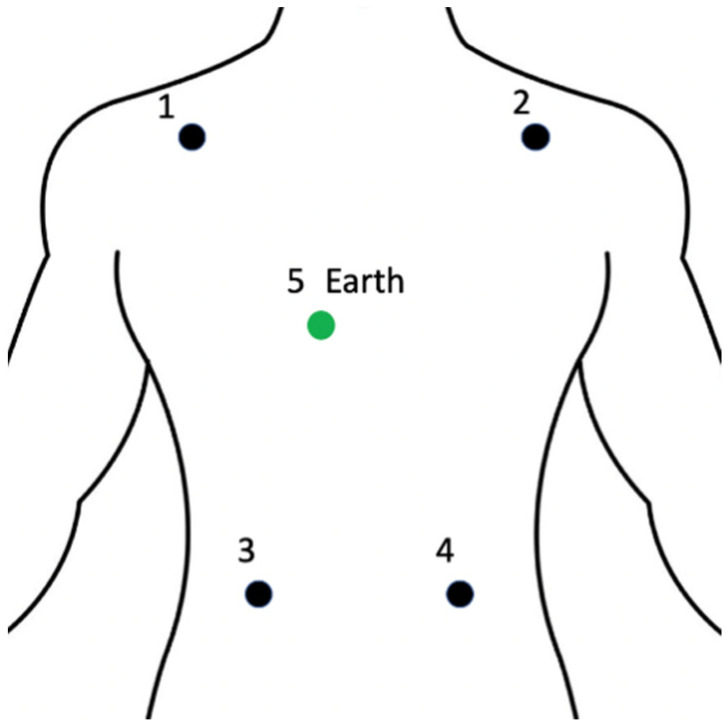
Standard 5-lead ECG placement layout.

The electrodes of the clinical ECG are placed according to standard 5-lead configuration, for which the first electrode is placed on the right side of the shoulder, the other electrode is on the left side of the shoulder, the third electrode is on the lower left side. The fourth electrode is on the lower right abdominal area and the earth electrode is on the right side of the sternum bottom, as illustrated in Figure 4.

To characterise the BR, a medical-grade and clinically validated device for respiratory sensing (ADInstrument spirometer) was used. The spirometer is a transducer of differential pressure to measure respiration flow rate, the volume and flow of inhaled and exhaled air. The sampling frequency for the breathing waveform is 100 Hz.

### 2.1. Performance Characterisation of Cardiorespiratory Measurements

The fundamental metrics adopted for benchmarking the performance of the commercial device are Root Mean Square Error (RMSE), standard deviation (σ), Correlation Coefficient (CC), and Mean Bias (MB) across the whole dataset. These metrics were selected as the most indicative of measurement validity and are defined as:

(1) $$RMSE=\sqrt{\frac{\sum_{i=1}^{n}{\left(s_{ti}-s_{oi}\right)}^{2}}{n}}$$

(2) $$\sigma =\sqrt{\frac{\sum_{i=1}^{n}{\left(d_{i}-\mu_{d}\;\right)}^{2}}{n}}$$

(3) $$CC=\frac{\mathrm{cov}\left(s_{t},s_{o}\right)}{\sigma_{s}{}_{t}\sigma_{s}{}_{o}}$$

(4) $$MB=\frac{\sum_{i}^{n}d_{i}}{n}$$

where *n* is the number of data points; $s_{t}$ is the data measured from commercial device in (1/min); $s_{o}$ is the data measured from clinical device in (1/min); d is the difference between $s_{t}$ and $s_{o}$ in (1/min); $\mu_{d}$ is the average difference between $s_{t}$ and $s_{o}$ in (1/min).

**Figure 5:**
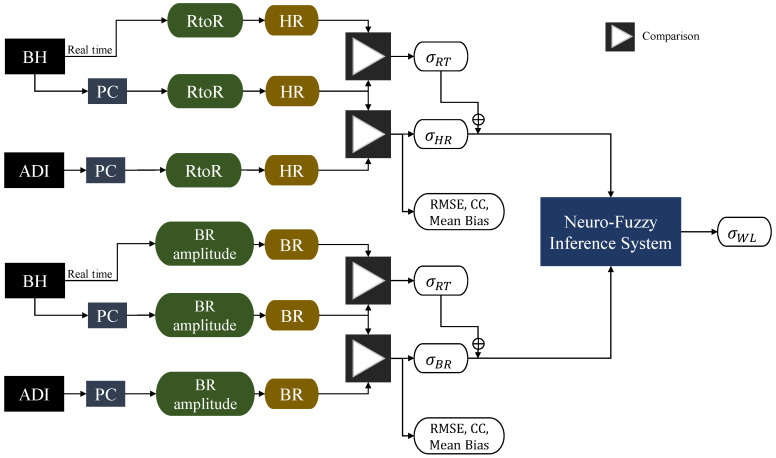
Performance analysis methodology for the wearable cardiorespiratory sensor validity.

Figure 5 illustrates the high-level methodology adopted throughout this performance characterisation of the commercial and clinical devices directly connected to a Personal Computer (PC). As illustrated, three different stages of the cardiorespiratory data processing are evaluated: accuracy and precision of offline-logged measurements, of real-time streamed data, and of cognitive estimates from the neuro-fuzzy inference system, since the cardiorespiratory data is used in determining the operator’s cognitive states in the estimation module. The HR is directly derived from the pulse-to-pulse (RtoR) interval, whereas the BR is derived from the raw breathing amplitude. Consequently, $\sigma_{HR}$ and $\sigma_{BR}$ in the figure are the uncertainties in logged commercial device data as compared to the clinical device (results provided in Section 3), whereas $\sigma_{RT}$ is the standard deviation calculated comparing real-time data to post-processing data (discussion provided in Section 2.2). Lastly, the uncertainty in estimated workload ($\sigma_{WL}$) is calculated since, as already mentioned, the purpose of real-time HR and BR measurements is to estimate mental workload and other cognitive states by means of a neuro-fuzzy inference system (results provided in Section 3.1). This part is vital since the inferred workload of the human operator is exploited in CHMI^2^. Hence, the measurement uncertainties of HR and BR affect the reliability of the workload.

### 2.2. Real Time Data Streaming and Processing Protocols

The integration of cardiorespiratory sensing as part of an adaptive system such as the CHMI^2^ requires real-time streaming and processing of the measurement data. To comply with this fundamental requirement, the performance of the commercial sensor in real-time measurement data communication were also assessed. For this functionality, the sensor allows real time data exchange via Bluetooth to any computing device also equipped with Bluetooth which can run a suitable setup and data processing software, with signal carrier frequency of 2.4–2.835 GHz [42]. Bluetooth is a well-established wireless communication technology utilising ultra-high frequency radio waves to exchange data between mobile devices, computers, and components over short distances. Bluetooth was standardised by IEEE 802.15.1 [43]. Bluetooth Special Interest Group (SIG), the international standards organisation for Bluetooth technology, regulates on hardware specifications and standardises devices sold in the market to ensure the devices meet the standard, so they can be shipped with attached licenses.

The same test process from Section 2.1 is also applied in this case, but this time comparing the real-time data with post-processing (offline) data. This test demonstrated that the two data samples are exactly consistent with 1 CC, zero RMS error, mean bias and σ. Hence, $\sigma_{RT}$ for both HR and BR is zero. However, we shall note that the Bluetooth signal is affected by packet losses and connection drops, particularly in presence of significant Electro-Magnetic Interference (EMI), when significant solid obstructions lie in the line-of-sight path between the sensor and the computing unit, or when the distance of the sensor from the computing unit is excessive, resulting in highly attenuated signals. These issues are commonly investigated by telecommunication specialists and several studies are available in the related literature. A specific characterisation of the Bluetooth link between the BH and the computing unit would not yield significantly different results as compared to the literature, with a possible exception in the case of a well-designed connection “watchdog” functionality having been implemented, leading to more consistent and timely restoration of dropped connections. The implementation of a suitable connection management interface such as *LabStreamingLayer* can allow to mitigate the effect of temporary connection drops without interrupting the data stream [44].

### 2.3. Propagation of Uncertainty across a Neuro-Fuzzy Inference Process

The uncertainty in the operator’s WL is estimated from cardiorespiratory and other physiological data by means of a neuro-fuzzy inference system, which is implemented as part of the CHMI^2^ to process the real-time cardiorespiratory measurements. Fuzzy systems provide some flexibility in adapting the system parameters to individual users so that the correlations exploited by the CHMI^2^ are unique to different individuals and their daily neurophysiological/mental state.

The methodology to derive the uncertainty in the classified cognitive state as a function of the neurophysiological inputs builds upon the initial studies we carried out in [14]. However, compared to that initial study, in this section we focus on the pre-clustering and training results based on experimental datasets and their effect on the uncertainty. In this study, HR and BR are the assumed inputs to the neuro-fuzzy inference system, and the output is estimated as WL. The fuzzy set is characterised by a set of fuzzy rules and membership functions. Fuzzy membership functions can assume various forms, which yield different advantages and disadvantages. Fundamental types include triangular, trapezoidal, gaussian, bell, and sigmoidal functions, which are described below. The gaussian membership function, used in our implementation, is defined by parameters (μ, σ) as:

(5) $$\delta \left(x\right)=exp\left[-\frac{{\left(x-\mu \right)}^{2}}{2\sigma^{2}}\right]$$

where δ is the degree of membership, μ is the centre of the membership function, and σ is the standard deviation of the cluster, which is correlated to the width of the membership function. The pre-clustering process is the first step of the neuro-fuzzy system calibration. The chosen initial clustering algorithm is Fuzzy C-Means (FCM) due to its consistent accuracy. The number of clusters are required to specify upfront. A membership matrix U is generated, which specifies whether data point $x_{i}$ belongs to group *j*. The sum of each data point’s membership must be unified across all groups [45]:

(6) $$\overset{c}{\underset{j=1}{\sum }}u_{ij}=1,\;\forall_{j}=1,\ldots ,n$$

Thereafter, a cost function is provided by

(7) $$J\left(U,\;c_{1},\ldots ,c_{c}\right)=\overset{c}{\underset{j=1}{\sum }}\overset{n}{\underset{i}{\sum }}u_{ij}{}^{m}\cdot d_{ij}{}^{2}$$

where $c_{j}$ is the centre of cluster group *j*, $u_{ij}$ is the degree of membership of data point *i* in group *j, m* is the weighting exponent which is a parameter that significantly effects the performance of the FCM, and $d_{ij}$ is defined by $\left\|x_{i}-c_{j}\right\|$ which is the Euclidean distance between the *i*-th data point and *j*-th cluster centre. The degree of fuzzy overlap is increased by increasing the value of *m*. The necessary conditions for minimising are given by:

(8) $$\begin{array}{cc} & J\left(U,\;c_{1},\ldots c_{c},\;\lambda_{1},\ldots \lambda_{n}\right)=J\left(U,\;c_{1},\ldots ,c_{c}\right)+\sum_{i}^{n}\lambda_{i}\cdot \left(\sum_{j=1}^{c}u_{ij}-1\right)\\ & =\sum_{j=1}^{c}\sum_{i}^{n}u_{ij}{}^{m}\cdot d_{ij}{}^{2}+\sum_{i}^{n}\lambda_{i}\cdot \left(\sum_{j=1}^{c}u_{ij}-1\right) \end{array}$$

The minimum of $J\left(U,\;c_{1},\ldots c_{c},\;\lambda_{1},\ldots \lambda_{n}\right)$ can be determined by differentiating it with respect to all input arguments. The necessary conditions are given by:

(9) $$c_{i}=\frac{\sum_{i}^{n}u_{ij}{}^{m}\cdot x_{i}}{\sum_{i}^{n}u_{ij}{}^{m}}$$

(10) $$u_{ij}=\frac{1}{\sum_{n=1}^{c}{\left(\frac{d_{ij}}{d_{kj}}\right)}^{2/\left(m-1\right)}\;}$$

The *c* cluster centres and m membership degrees are initialised arbitrarily and are subsequently updated using Equations (7) and (8) respectively, followed by computing an updated *J* from Equation (6). The iteration of the degree of membership and cluster centres calculation is advanced until *J* satisfies a given threshold or until $\left\|U^{\left(k+1\right)}-U^{\left(k\right)}\right\|$ satisfies a termination criterion. Subsequently, the second phase involves the process of calibrating the generated fuzzy cluster parameters, thus tuning them to maximise the correlations between inputs and output(s). We note that the Adaptive Neuro-Fuzzy Inference System (ANFIS) framework available as a MATLAB library and adopted for the majority of the work presented in this article only allows one individual output to be included. Hybrid training method is selected to adjust the input membership function (cluster) parameters due to superior performance, while the function parameters of the output membership are used in the training phase. The type of chosen ANFIS [46] is *K* Takagi-Sugeno, with rules mapping input to output formulated as

$$\mathrm{Rule}\;k\text{:}\;\mathrm{If}\;x_{1}\;\mathrm{is}\;A_{1n}\;\mathrm{and}\;x_{2}\;\mathrm{is}\;A_{2n}\;\mathrm{and}\;...\;\mathrm{and}\;x_{i}\;\mathrm{is}\;A_{in}\;\mathrm{then}$$

f_j_ = p_k0_ + p_k1_ x_1_ + p_k2_ x_2_ + … + p_ki_ x_i_

where $A_{in}$ is the nth input $x_{i}$ membership function, $f_{j}$ is the node output function of output j, and $p_{kj}$ denotes the coefficients for rule k and input i of this node function.

The WL is assumed to be linearly correlated to level of difficulty. Low HR and BR represent low workload, while high HR and BR represent high workload [47,48]. Therefore, the rule-base consists of two fuzzy rules:

If HR is low and BR is low, then WL = 0.098 + 0.1167x_1_ − 9.8x_2_;

If HR is high and BR is high, then WL = 0.123 − 0.016x_1_ − 9.8 x_2_.

WL is normalised value from zero to one; low is 0.3 and high is 0.7, because this LOD of the math exercise does not require 100% of their cognitive capability based on the subjective rating. The methodology proposed and evaluated in [14] considered the shape and distribution of the membership functions as determined by the training process. It did not introduce any assumption regarding the order or shape of the psychophysiological response curve. Because of this, intervals in the physiological input data for which no training data was available (hence not covered by any membership function) led to very significant penalties (i.e., higher uncertainty). However, it was excessively penalising in terms of uncertainty when considering that the human psychophysiological response from the reference are known to be very smooth and low-ordered [34] so that it can be assumed that no peak or trough occurs in the interval for which no input training data is provided. We therefore propose here a new approach that is based on the assumption of a smooth low-order psychophysiological response curve. Although training input data was not available for all intervals, based on the literature we can safely assume this smooth surface without a large jump to be reasonably close to the real human response [49]. With this assumption, we can directly use the propagation of uncertainty methodology to estimate the uncertainty in WL. For any nonlinear differentiable function f, the generic formulation of the uncertainty propagation is derived from the following multivariate expansion:

(11) $$\sigma_{f}^{2}=\underset{i=1,n}{\sum }\left[{\left(\frac{\partial f}{\partial x_{i}}\sigma_{x_{i}}\right)}^{2}+\underset{j=1,n;\;i\neq j}{\sum }\left(2\frac{\partial f}{\partial x_{i}}\frac{\partial f}{\partial x_{j}}\;\sigma_{x_{i}x_{j}}\right)\right]$$

where $x_{i}$ are the independent variables. Hence, the uncertainty propagation of the WL estimates as a function of the uncertainty in the physiological input uncertainties takes the following form:

(12) $$\sigma_{WL}^{2}={\left(\frac{\partial WL}{\partial HR}\right)}^{2}\sigma_{HR}^{2}+{\left(\frac{\partial WL}{\partial BR}\right)}^{2}\sigma_{BR}^{2}+2\frac{\partial WL}{\partial HR}\frac{\partial WL}{\partial BR}\sigma_{HRBR}$$

where $\sigma_{WL}^{2}$ is the variance in the workload estimate, $\sigma_{HR}^{}$ is the variance in HR, $\sigma_{BR}$ is the variance in BR, $\sigma_{HRBR}$ is the covariance term of HR and BR (determined based on the measurement population).

The process to derive a polynomial surface from a FIS was mathematically discussed in [46,50,51] and is implemented as part of the MATLABs command «gensurf».

### 2.4. Experiment Design and Raw Measurement Data Processing

**Figure 6:**
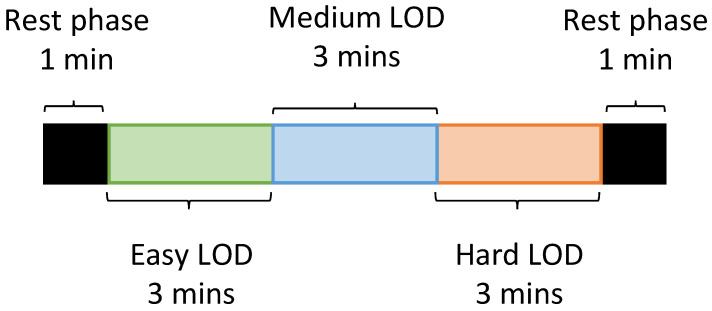
Experimental protocol of the mental workload exercise.

All the adopted research methods and data collection protocols were approved by RMIT’s University College Human Ethics Advisory Network (CHEAN) (ref: ASEHAPP 72-16) and all participants provided written consent. The experiment involves ten participants (eight males, two females, age: 28 ± 4.8 years). The experiments were held in the late morning for all participants. The tasks required each participant to complete basic math calculations that varied in three Levels of Difficulty (LOD) for three minutes at each level: easy, medium, and high. At each level, every question included addition, subtraction, multiplication, and division. The difficulty increased by adding more digits in medium and high level. In addition, the time limit for each question also varies among each level: 60 s for easy, 40 s for medium, and 30 s for hard. The one-minute rest state was measured before and after the test, as illustrated in Figure 6. Mathematical calculations were chosen over more realistic ATM exercises as they have been shown to stimulate demanding levels of mental workload [52] while having the flexibility of not requiring prior ATM experience from participants.

Upon completion of the above-mentioned testing protocols, raw ECG signals were extracted from both commercial and clinical devices. Then, the R peaks of the signal were identified to calculate instantaneous HR. After identification of each peak, the R to R interval (RtoR) can be calculated by the taking the time difference between consecutive peaks, which is used to calculate HR, as:

(13) $$HR\;\;\left[bpm\right]=\frac{60}{RtoR\;\;\left[s\right]}$$

To compare the *HR* signal, the clock and sampling rate of the two sensors were collected and processed separately, as they can differ. The time was scaled to ensure the starting and finishing time of two datasets are synchronised, and the ECG signal obtained from the clinical device was down-sampled from 1000 Hz to 250 Hz. Moreover, the datasets have been resampled to make sure that the data from both sensors are synchronised. Although there are various sources of electromagnetic noise in the targeted application environment, the experiments we conducted were carried out in a very representative setting and no significant electromagnetic interference was detected. However, there is still considerable noise in the data (i.e., movement artefacts), which can affect the validity of the system. Hence, before going through system performance analysis, the *HR* signal was filtered, as in most practical cardiorespiratory monitoring applications. The chosen process is low-pass filtering of 2nd order Butterworth type, which is arguably the most consistent and repeatable raw signal processing technique for removing high frequency noise. This filter lets through signals lower than a selected cut-off frequency and lessens signals higher than the cut-off frequency. Such low-pass filtering smooths the data and is specifically tuned to increase the accuracy of the measurement considering the physics and physiological specificities of the monitored bio-signal. For instance, HRV is an important feature in the CHMI^2^ framework [13] and can be divided in two distinct bands: Low-Frequency (LF HRV), which spans between 0 Hz and 0.15 Hz, and High-Frequency (HF HRV) which spans between 0.15 Hz and 0.4 or 0.45 Hz [53]. As both physical and mental WL are mainly correlated with LF HRV, for our particular application the cut-off frequency could have been set as low as 0.15 Hz, however such strict filtering would have unnecessarily restricted the sensor characterisation to LF HR/HRV monitoring. Therefore, to support a conservative characterisation of the sensor for all HRV components and to limit the amount of filtered data, the cut-off frequency was set to 1 Hz with steepness of 0.85, which is more than twice the maximum physiological HF HRV component. Since HRV is directly derived from the same RtoR as *HR*, a single performance characterisation is presented which is applicable to both cardiac signals. Finally, a data rejection policy was also adopted such that *HR* values lower than 50 bpm and higher than 180 bpm, and BR values lower than 5 bpm and higher than 30 bpm, were discarded.

The breathing waveforms from the commercial and clinical devices were extracted and the associated BR was calculated. In particular, the waveform was differentiated in time and the positive and negative peaks in the BR derivative were identified to indicate inhaling and exhaling, respectively. The BR was subsequently calculated for both datum and measured breathing in the same manner as Equation (1), with the exception that the RtoR is replaced by the time difference between onsets of inhaling events (peaks in the first derivative of the chest expansion magnitude signal). The results were up-sampled to a common time reference and subsequently a low-pass filtering was introduced with cut-off frequency of 1 Hz with a steepness of 0.95. This filtering is at least as equally conservative as the HRV one discussed above, as the human breathing has a lower frequency than the heartbeat signal.

## 3. Sensor Characterisation Results

**Figure 7:**
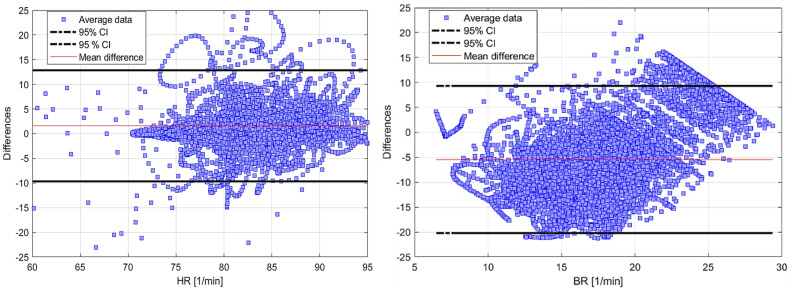
Bland–Altman plots of HR (**left**) and BR (**right**) for the entire population. The mean differences (red line) and the 95% confidence intervals (black lines) are also shown.

Table 1 presents the aggregated validity results of the commercial device compared to the baseline clinical sensor measurements, after the application of data rejection criteria described in Section 2.4. For HR, the average RMSE across all participants is 4.852 bpm and the average CC is 0.663. Looking only at aggregated correlations between two datasets has some limitations: the discrepancy indication is not given, and the agreement is not fully assessed. Therefore, the Bland–Altman plot is also adopted to further analyse the characterisation results. Such a plot shows the values scatter, values ranges, the systematic difference level, random errors, a relation between two protocols, and importantly the result variations [54]. Figure 7 presents the Bland–Altman plots for the entire dataset, separately in terms of HR (left) and BR (right). Each blue dot represents the average measurement error (difference between the commercial and clinical sensors) while the black lines represent 95% confidence interval or limits of agreement. The range of values can be easily visualised, whether they are small or large, from these limits of agreement lines.

**Figure 8:**
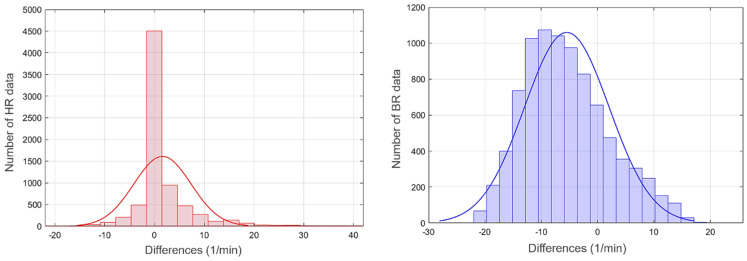
Statistical distributions (histograms) of HR (**left**) and BR (**right**) errors for the entire population. The normal (Gaussian) fit curve is also plotted for reference.

The final step of the sensor characterisation involved looking at the statistical distributions of measurement errors. The HR and BR measurement error distributions for all participants are presented as histograms in Figure 8. The figure also represents the normal distribution (Gaussian) fits. In the BR case, compared to HR, there is a higher mean bias (µ) and σ, which makes BR a right-skewed distribution.

### 3.1. Uncertainty in the Inference System

**Figure 9:**
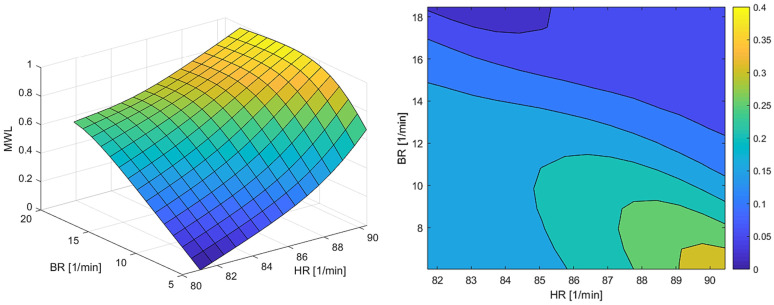
(**Left**) psychophysiological response surface for one of the participants. (**Right**) Uncertainty in WL as propagated through the psychophysiological response surface.

Using the polynomial coefficients from the psychophysiological response surface (illustrated in Figure 9 left below), the uncertainty in WL estimates can be determined for any value of HR and BR. The results for a particular participant, given sensor uncertainties calculated from the previous section of $\sigma_{HR}$ = 0.720, $\sigma_{BR}$ = 6.534 and $\sigma_{HRBR}$ = −0.560, are depicted in Figure 9 (right).

Table 2 depicts the results from all participants, and the sensor uncertainties are applied to each dataset.

## 4. Discussion of Results

**Figure 10:**
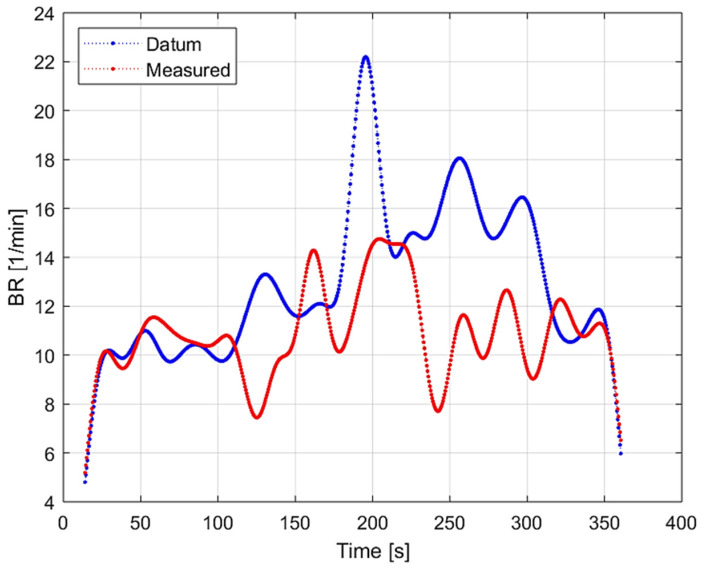
Comparison of filtered BR time series from clinical (blue line) and commercial device (red line).

From the characterisation of raw cardiorespiratory measurements (Table 1), the CC in HR was above 0.50 for the vast majority of cases, which indicates a moderate (or better) correlation. The minimum mean bias in HR is 1.511 bpm, which is very low, while the maximum is 10.48 bpm. The maximum errors for HR are all from one participant that appeared to wear the loosened strap. In particular, it was observed that the HR data from one participant and BR data from the same and another participant were not correctly detected by the commercial monitor, most likely because of the incorrectly tightened strap. As this is an acknowledged limitation of the sensor, documented in the manual, the data from these particular participants should have been rejected, but it was nevertheless included in the results for completeness. The BR performance was consistently worse compared to HR. In particular, the correlation between BR signals was poor overall, as indicated by the CC. Moreover, the BR results also showed large RMSE, σ and mean bias with a value of −9.729, 7.394, and −6.003 bpm, respectively. From the Bland–Altman plots (Figure 7), rather large variations of HR and BR are visible. In particular, Figure 7 (left) highlighted that the mean difference increased in both positive and negative directions at higher values of HR. The mean differences across participants was 3.021 bpm. The maximum differences between the commercial and clinical devices was ±25 which is consistent with [17]. Figure 7 (right) also showed a trend of increasing negative difference at higher BR values. Finally, the Bland–Altman plots highlighted that there were no significant differences across participants, confirming the correctness of low CC results for BR. The mean BR difference among participants is −5.466 bpm, which is higher than the value quoted in the technical specifications. Further analysis of these poor results required us to inspect the raw BR time series (Figure 10 below) and the respiratory magnitude plots for all participants. From these further investigations, we could conclude that the commercial device systematically missed breaths of smaller amplitude and more frequent occurrence.

Looking at the uncertainty in the estimated cognitive workload (Section 3.1), the assumed acceptable range of $\sigma_{WL}$ was ±45%. Although apparently high, this level is reasonable considering that the cardiorespiratory response is much slow than the cognitive processes that we are estimating [55], and also because the activities that we administered to the participants were likely lower than the maximum cognitive load that they could withstand. Attempting to test the whole cognitive range would have required an adaptive exercise, which will be considered for future research. From Figure 9 (right), it is evident that for a wide range of HR and BR inputs, the uncertainty is relatively low (below 25%), however it increases notably in a small region of high HR and low BR. The best uncertainty in inferred workload $\sigma_{WL}$ is 37.64% (Table 2). The worst case of maximum uncertainty in inferred workload is 222%, clearly due to a very high uncertainty from BR. When such large uncertainties are determined, the calibration process of the cardiorespiratory device should be re-conducted until the acceptable error is achieved before starting real-time measurement. Table 3 presents the uncertainty in inferred workload using only HR as input variable, as we clearly identified that the high uncertainty in BR causes significant errors in the estimation process as well. The average $\sigma_{WL}$ decreases by 38.96% from 1.329 to 0.811. The worst case shows improved performance but is still unusable. The average $\sigma_{WL}$ without the worst case is decreased to 0.356 from 0.811, which falls within the acceptable range less than 0.45.

The overall conclusion of the verification is that the measurement validity of BR is inadequate for mental activity monitoring, where smaller and more frequent breaths are not uncommon. It shall nonetheless be reiterated that the BH was specifically designed for sport and sport medicine applications, so these findings are not in contrast with the expectations. Based on the results of this characterisation, alternative respiratory sensors need to be considered for the CHMI^2^ implementation. Hemodynamic sensors, which sense variables associated with the blood flow, hold particular promise, as some of these are less intrusive and potentially more accurate in mental activity monitoring. Their measurement validity will be analysed as part of future research.

## 5. Conclusions

This article addressed the experimental characterisation of a commercial cardiorespiratory sensor for emerging Air Traffic Management (ATM) and avionics Human–Machine Systems (HMS) applications. Cognitively complex tasks of this type are associated with high mental workload (WL), however no previous research addressed the verification and performance analysis of commercial sensors which are commonly used in these mental workload studies. The commercial sensor subject to this study is a commonly used wearable consumer-grade device for sport and sport medicine applications, capable of both offline logging and real-time data streaming of raw and processed cardiorespiratory data. The measurement validity and accuracy of both Heart Rate (HR) and Breathing Rate (BR) measurements from the wearable commercial device were assessed by direct comparison with a clinically validated device during representative mental workload exercises.

The article also presented and applied a novel methodology to quantify the uncertainty in the cognitive state estimates based on the uncertainty in input physiological data, expanding the traditional propagation of uncertainty theory. In particular, the uncertainty in WL and other cognitive states estimates was quantified from the cardiorespiratory measurements, propagating these through the psychophysiological response surface which was determined by the neuro-fuzzy inference system. The analysis showed that the commercial device achieved good accuracy in cardiac (HR) measurements but performed poorly in terms of BR measurement during mental workload exercises. Consequently, the uncertainty in the cognitive state estimates was acceptable only if limited to the cardiac measurements. Based on this verification activity, the selected device is adequate for cardiac monitoring as part of the targeted aerospace HMS application, but alternative devices will have to be considered for respiratory monitoring. The main candidates are hemodynamic sensors, which are typically less intrusive and potentially more accurate in mental activity monitoring. This work contributes to the broader research on Cognitive Human-Machine Interfaces and Interactions (CHMI^2^) for ATM and avionics applications, which is one of the key areas of aerospace systems innovation. Further research will look at the integration and optimal fusion of various neurophysiological sensors to accurately monitor the cognitive states in complex tasks.

## Figures and Tables

**Table 1:** HR and BR validity results for the commercial device.

	RMSE [1/min]	σ [1/min]	CC	Mean Bias [1/min]
HR	4.852	4.109	0.663	1.901
HR min error	0.728	0.720	0.990	−1.511
HR max error	14.86	10.55	0.319	10.48
BR	−9.729	7.394	0.087	−6.003
BR min error	−7.958	6.534	0.188	−2.771
BR max error	−12.94	8.024	0.029	−15.80

**Table 2:** $\sigma_{HR}$, $\sigma_{BR}$, $\sigma_{HRBR}$, and $\sigma_{WL}$ from the neuro-fuzzy inference system.

	$\sigma_{HR}$	$\sigma_{BR}$	$\sigma_{HRBR}$	$\sigma_{WL}$
Best	0.720	6.534	−0.560	0.376
Worst	2.491	7.433	17.49	2.220
Average	4.109	7.394	0.850	1.329

**Table 3:** $\sigma_{HR}$ and $\sigma_{WL}$ from the neuro-fuzzy inference system.

	$\sigma_{WL}$	% Decrease
Best	0.125	33.39
Worst	1.211	45.45
Average	0.811	38.96

## Data Availability

The data presented in this study are not publicly available due to the confidentiality clauses of the above mentioned CHEAN ethics approval data management.

## References

[1] Jeannot E., Kelly C., Thompson D. (2003). The Development of Situation Awareness Measures in ATM Systems. EATMP Rep. https://www.semanticscholar.org/paper/The-Development-of-Situation-Awareness-Measures-in-Jeannot-Thompson/fea68619b429499881bb42ebddd5e0f652346f33.

[2] Sulistyawati K., Wickens C.D., Chui Y.P. (2011). Prediction in Situation Awareness: Confidence Bias and Underlying Cognitive Abilities. Int. J. Aviat. Psychol..

[3] Lim Y., Gardi A., Sabatini R., Ramasamy S., Kistan T., Ezer N., Vince J., Bolia R. (2018). Avionics Human-Machine Interfaces and Interactions for Manned and Unmanned Aircraft. Prog. Aerosp. Sci..

[4] Borghini G., Aricò P., Di Flumeri G., Cartocci G., Colosimo A., Bonelli S., Golfetti A., Imbert J.P., Granger G., Benhacene R. (2017). EEG-Based Cognitive Control Behaviour Assessment: An Ecological study with Professional Air Traffic Controllers. Sci. Rep..

[5] Mansikka H., Simola P., Virtanen K., Harris D., Oksama L. (2016). Fighter pilots’ heart rate, heart rate variation and performance during instrument approaches. Ergonomics.

[6] Sompagnimdi M.T., Hurter C. Exploratory Study with Eye Tracking Devices to Build Interactive Systems for Air Traffic Controllers. Proceedings of the HCI-Aero ‘16: International Conference on Human-Computer Interaction in Aerospace.

[7] Amos B., Ludwiczuk B., Satyanarayanan M. (2016). Openface: A general-purpose face recognition library with mobile applications. CMU Sch. Comput. Sci..

[8] Fydanaki A., Geradts Z. (2018). Evaluating OpenFace: An open-source automatic facial comparison algorithm for forensics. Forensic Sci. Res..

[9] Fayek H.M., Lech M., Cavedon L. (2017). Evaluating deep learning architectures for Speech Emotion Recognition. Neural Netw..

[10] Vukovic M., Sethu V., Parker J., Cavedon L., Lech M., Thangarajah J. (2019). Estimating cognitive load from speech gathered in a complex real-life training exercise. Int. J. Hum. Comput. Stud..

[11] Huang Z., Dong M., Mao Q., Zhan Y. Speech Emotion Recognition Using CNN. Proceedings of the 22nd ACM International Conference on Multimedia.

[12] Lim W., Jang D., Lee T. Speech emotion recognition using convolutional and Recurrent Neural Networks. Proceedings of the 2016 Asia-Pacific Signal and Information Processing Association Annual Summit and Conference (APSIPA).

[13] Pongsakornsathien N., Lim Y., Gardi A., Hilton S., Planke L., Sabatini R. (2019). Sensor Networks for Aerospace Human-Machine Systems. Sensors.

[14] Lim Y., Gardi A., Pongsakornsathien N., Sabatini R., Ezer N., Kistan T. (2019). Experimental characterisation of eye-tracking sensors for adaptive human-machine systems. Measurement.

[15] Nunan G.D., Donovan D.G., Jakovljevic R.H.D., Hodges A.L., Sandercock A.G., Brodie A.D. (2009). Validity and Reliability of Short-Term Heart-Rate Variability from the Polar S810. Med. Sci. Sports Exerc..

[16] Hailstone J., Kilding A.E. (2011). Reliability and Validity of the Zephyr BioHarness to Measure Respiratory Responses to Exercise. Meas. Phys. Educ. Exerc. Sci..

[17] Johnstone J.A., Ford P.A., Gerwyn H., Watson T., Garrett A.T. (2012). BioHarness Multivariable Monitoring Device: Part I: Validity. J. Sports Sci. Med..

[18] Johnstone J.A., Ford P.A., Hughes G., Watson T., Garrett A.T. (2012). Bioharness Multivariable Monitoring Device: Part II: Reliability. J. Sports Sci. Med..

[19] Smith D.L., Haller J.M., Dolezal B.A., Cooper C.B., Fehling P.C. (2013). Evaluation of a Wearable Physiological Status Monitor during Simulated Firefighting Activities. J. Occup. Environ. Hyg..

[20] Flanagan D.S., Comstock A.B., Dupont H.W., Sterczala R.A., Looney P.D., Dombrowski H.D. (2014). Concurrent Validity of the Armour39 Heart Rate Monitor Strap. J. Strength Cond. Res..

[21] Dolezal B., Boland D., Carney J., Abrazado M., Smith D., Cooper C. (2014). Validation of Heart Rate Derived from a Physiological Status Monitor-Embedded Compression Shirt against Criterion ECG. J. Occup. Environ. Hyg..

[22] Rawstorn J.C., Gant N., Warren I., Doughty R.N., Lever N., Poppe K.K., Maddison R. (2015). Measurement and Data Transmission Validity of a Multi-Biosensor System for Real-Time Remote Exercise Monitoring Among Cardiac Patients. JMIR Rehabil. Assist. Technol..

[23] Nazari G., Bobos P., MacDermid J.C., Sinden K.E., Richardson J., Tang A. (2018). Psychometric properties of the Zephyr bioharness device: A systematic review. BMC Sports Sci. Med. Rehabil..

[24] Galli A., Narduzzi C., Giorgi G. (2018). Measuring Heart Rate During Physical Exercise by Subspace Decomposition and Kalman Smoothing. IEEE Trans. Instrum. Meas..

[25] Nazari G., Macdermid J.C., Kin K.E.S.R., Richardson J., Tang A. (2019). Reliability of Zephyr Bioharness and Fitbit Charge Measures of Heart Rate and Activity at Rest, During the Modified Canadian Aerobic Fitness Test and Recovery. J. Strength Cond. Res..

[26] Kaber D.B., Perry C.M., Segall N., Sheik-Nainar M.A. (2007). Workload State Classification with Automation During Simulated Air Traffic Control. Int. J. Aviat. Psychol..

[27] Vogt J., Hagemann T., Kastner M. (2006). The Impact of Workload on Heart Rate and Blood Pressure in En-Route and Tower Air Traffic Control. J. Psychophysiol..

[28] Taj-Eldin M., Ryan C., O’Flynn B., Galvin P. (2018). A Review of Wearable Solutions for Physiological and Emotional Monitoring for Use by People with Autism Spectrum Disorder and Their Caregivers. Sensors.

[29] Wilson G.F. (2002). An Analysis of Mental Workload in Pilots During Flight Using Multiple Psychophysiological Measures. Int. J. Aviat. Psychol..

[30] Bonner M.A., Wilson G.F. (2002). Heart Rate Measures of Flight Test and Evaluation. Int. J. Aviat. Psychol..

[31] Lahtinen T., Koskelo J.P., Laitinen T., Leino T. (2007). Heart rate and performance during combat missions in a flight simulator. Aviat. Space Environ. Med..

[32] Hankins T.C., Wilson G.F. (1998). A comparison of heart rate, eye activity, EEG and subjective measures of pilot mental workload during flight. Aviat. Space Environ. Med..

[33] Pongsakornsathien N., Gardi A., Lim Y., Sabatini R., Kistan T., Ezer N. Performance Characterisation of Wearable Cardiac Monitoring Devices for Aerospace Applications. Proceedings of the IEEE International Workshop on Metrology for AeroSpace (MetroAeroSpace).

[34] Lim Y., Ramasamy S., Gardi A., Kistan T., Sabatini R. (2018). Cognitive Human-Machine Interfaces and Interactions for Unmanned Aircraft. J. Intell. Robot. Syst..

[35] Liu J., Gardi A., Ramasamy S., Lim Y., Sabatini R. (2016). Cognitive pilot-aircraft interface for single-pilot operations. Knowl. Based Syst..

[36] Ivonin L., Chang H.-M., Chen W., Rauterberg M. Automatic recognition of the unconscious reactions from physiological signals. Proceedings of the International Conference on Human Factors in Computing and Informatics.

[37] Bradley M.M., Miccoli L., Escrig M.A., Lang P.J. (2008). The pupil as a measure of emotional arousal and autonomic activation. Psychophysiology.

[38] Kwon O., Jeong J., Kim H.B., Kwon I.H., Park S.Y., Kim J.E., Choi Y. (2018). Electrocardiogram Sampling Frequency Range Acceptable for Heart Rate Variability Analysis. Healthc. Inform. Res..

[39] Kim J.H., Roberge R., Powell J.B., Shafer A.B., Williams J.W. (2013). Measurement accuracy of heart rate and respiratory rate during graded exercise and sustained exercise in the heat using the Zephyr BioHarness. Int. J. Sports Med..

[40] Biopac Systems Product Sheet—BioHarness Data Logger and Telemetry Physiology Monitoring System, Datasheet. https://www.biopac.com/wp-content/uploads/BioHarness-BT.pdf.

[41] Simonetta G., Aziz N., Forrester K. (2006). Recent developments in data recording systems for Physiology. Pak. J. Physiol..

[42] (2015). Standard for Low-Rate Wireless Networks.

[43] (2015). Part 15.1: Wireless Medium Access Control (MAC) and Physical Layer (PHY) Specifications for Wireless Personal Area Networks (WPANs).

[44] Kothe C. Lab streaming Layer (LSL). Proceedings of the IEEE International Conference on Systems, Man and Cybernetics (SMC).

[45] Miyamoto S.A., Ichihashi H., Honda K. (2008). Algorithms for Fuzzy Clustering Methods in c-Means Clustering with Applications.

[46] Jang J.-S.R. (1993). ANFIS: Adaptive-network-based fuzzy inference system. IEEE Trans. Syst. Man Cybern..

[47] Fallahi M., Motamedzade M., Heidarimoghadam R., Soltanian A.R., Miyake S. (2016). Effects of mental workload on physiological and subjective responses during traffic density monitoring: A field study. Appl. Ergon..

[48] Marinescu A.C., Sharples S., Ritchie A.C., Lopez T.S., McDowell M., Morvan H.P. (2018). Physiological parameter response to variation of mental workload. Hum. Factors.

[49] Avram R., Tison G.H., Aschbacher K., Kuhar P., Vittinghoff E., Butzner M., Runge R., Wu N., Pletcher M.J., Marcus G.M. (2019). Real-world heart rate norms in the Health eHeart study. NPJ Digit. Med..

[50] Jang J.-S.R., Sun C.-T. (1995). Neuro-fuzzy modeling and control. Proc. IEEE.

[51] Jang J.-S.R., Sun C.-T. (1997). Neuro-Fuzzy and Soft Computing: A Computational Approach to Learning and Machine Intelligence.

[52] Tato A., Nkambou R., Ghali R. Towards Predicting Attention and Workload During Math Problem Solving. Proceedings of the International Conference on Intelligent Tutoring Systems.

[53] Shaffer F., Ginsberg J.P. (2017). An Overview of Heart Rate Variability Metrics and Norms. Front. Public Health.

[54] Van Stralen K.J., Jager K.J., Zoccali C., Dekker F.W. (2008). Agreement between methods. Kidney Int..

[55] Vlemincx E., Taelman J., de Peuter S., van Diest I., van den Bergh O. (2010). Sigh rate and respiratory variability during mental load and sustained attention. Psychophysiology.

